# Alternative Splicing: A New Cause and Potential Therapeutic Target in Autoimmune Disease

**DOI:** 10.3389/fimmu.2021.713540

**Published:** 2021-08-17

**Authors:** Pingping Ren, Luying Lu, Shasha Cai, Jianghua Chen, Weiqiang Lin, Fei Han

**Affiliations:** ^1^ Kidney Disease Center, The First Affiliated Hospital, Zhejiang University School of Medicine, Hangzhou, China; ^2^ Key Laboratory of Nephropathy, Zhejiang Province, Hangzhou, China; ^3^ Institute of Nephropathy, Zhejiang University, Hangzhou, China; ^4^ Department of Nephrology, The First People’s Hospital of Wenling, Taizhou, China; ^5^ Institute of Translational Medicine, Zhejiang University of Medicine, Hangzhou, China

**Keywords:** alternative splicing, autoimmune disease, systemic lupus erythematosus, rheumatoid arthritis, therapy

## Abstract

Alternative splicing (AS) is a complex coordinated transcriptional regulatory mechanism. It affects nearly 95% of all protein-coding genes and occurs in nearly all human organs. Aberrant alternative splicing can lead to various neurological diseases and cancers and is responsible for aging, infection, inflammation, immune and metabolic disorders, and so on. Though aberrant alternative splicing events and their regulatory mechanisms are widely recognized, the association between autoimmune disease and alternative splicing has not been extensively examined. Autoimmune diseases are characterized by the loss of tolerance of the immune system towards self-antigens and organ-specific or systemic inflammation and subsequent tissue damage. In the present review, we summarized the most recent reports on splicing events that occur in the immunopathogenesis of systemic lupus erythematosus (SLE) and rheumatoid arthritis (RA) and attempted to clarify the role that splicing events play in regulating autoimmune disease progression. We also identified the changes that occur in splicing factor expression. The foregoing information might improve our understanding of autoimmune diseases and help develop new diagnostic and therapeutic tools for them.

## Introduction

Alternative splicing is a vital mechanism in gene modulation. It allows a single gene to produce multiple distinct mRNA that greatly increase transcriptome and proteome diversity. According to recent genome-wide association studies (GWAS), nearly 95% of all protein-coding genes in the human genome undergo alternative splicing to varying degrees (1, 2). In this way, they increase by more than 10-fold of the number of distinct protein isoforms that function in various cellular activities, such as maturation, differentiation, migration, and death (3).

Alternative splicing is highly controlled. It comprises splice site selection, spliceosome assembly, and the control of multiple splicing regulatory elements. Any disruptions or mutations in the splicing mechanisms may affect mature mRNA and functional protein generation and induce various disease states. Over the last decade, particular attention has been allocated to disease-related genomics, transcriptomics, and proteomics. Alternative splicing might be associated with susceptibility to cancers, cardiovascular diseases, and different neuropathies, such as Alzheimer’s, Parkinson’s, and Huntington’s diseases as well as schizophrenia and congenital myasthenic syndrome. It is also related to infection, inflammation, and immune and metabolic disorders (4).

Autoimmune diseases include a wide range of disorders arising from an abnormal immune response to otherwise healthy, normal tissues and organs. Genetic, environmental, hormonal, and immunological etiologies are implicated in autoimmune disorder pathogenesis. However, the mechanisms that trigger the onset of at least half of all autoimmune diseases remain unclear (5). Numerous studies have investigated the roles of alternative splicing in autoimmunity mechanisms. Immune-related genes encoding human leukocyte antigen (HLA), interferon regulatory factor 5 (IRF5), T-cell receptor ζ (TCRζ) chain, cytotoxic T lymphocyte associated protein 4 (CTLA4), and cytokines and their receptors presented with spliced isoforms (6). However, our knowledge of the regulatory mechanisms of alternative splicing in immunity and autoimmunity is limited.

## Alternative Splicing Mechanism

### Splicing Reaction

**Figure 1:**
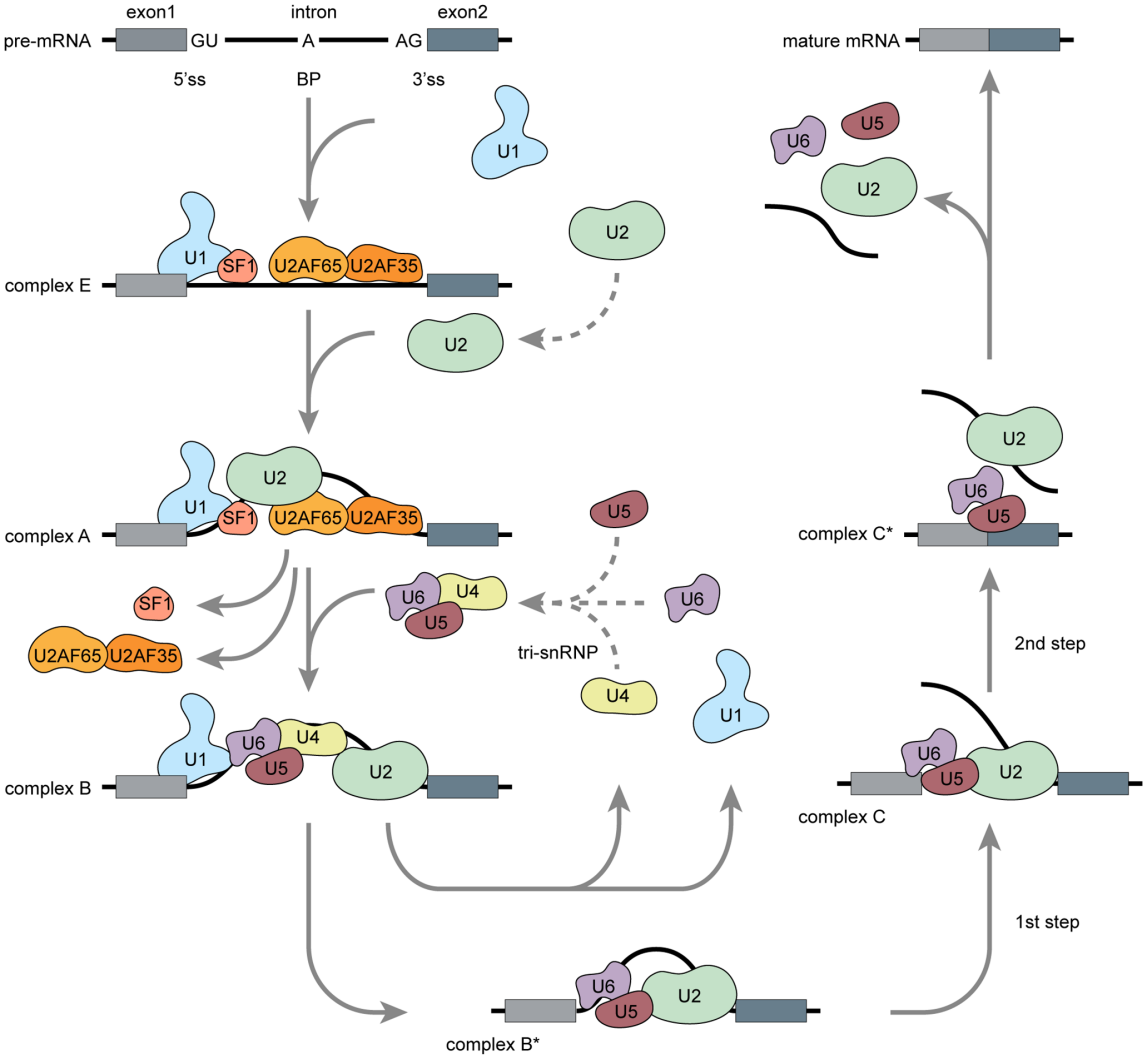
Pre-mRNA splicing: Spliceosome assembly (Adapted from Matera AG, Wang Z. A day in the life of the spliceosome. *Nat Rev Mol Cell Biol* (2014) 15:108-21). Pre-mRNA splicing occurs in several spliceosome assembly steps. Splicing begins with U1 snRNP assembly onto pre-mRNA *via* 5’ss recognition and subsequent combination with U2AF and SF1 to form early complex E. Then, U2 snRNP replaces SF1 at the branch point and forms a pre-spliceosome (complex A). Pre-assembled U4/U6–U5 tri-snRNP complexes are then recruited to form catalytically active complex B, which is then converted into catalytically active complex B* *via* release of U1 and U4. Through two catalytic steps and conformation or composition rearrangements, complex B* converted into a final complex C* and U2, U5, and U6 are released from the spliceosome followed by intron excision, exon ligation, and mature mRNA generation.

Precursor (pre) mRNA splicing is an intricate biological process involving intron excision and exon ligation to generate mature mRNA products. Splicing recognizes exon–intron boundaries and is catalyzed and controlled by complex ribonucleoprotein complexes known as spliceosomes (7). The spliceosome consists of at least 300 associated proteins and five uracil-rich small nuclear ribonucleoproteins (U1, U2, U4, U5, and U6 snRNPs) (8). Proper spliceosome assembly at the splice sites (ss) is the core reaction mechanism. The 5’ss is located at the 5’ end of each intron, the 3’ss is located at the 3’ end of each intron, and the branch point (BP) site is located 18–40 nucleotides upstream of each 3’ss (9). Spliceosome assembly begins with adenosine triphosphate (ATP)-independent recognition of the 5’ss by U1 snRNP. U2 auxiliary factor (U2AF) then binds the 3’ss and the polypyrimidine region. Splicing factor 1 (SF1) then binds the BP (**Figure 1**) (8, 10). These steps form the stable early complex E, which then triggers ATP-dependent U2 snRNP recruitment, SF1 replacement at the BP, and conversion into the pre-spliceosome (complex A) (10). The pre-assembled U4/U6–U5 tri-snRNP complex is recruited and forms a pre-catalytic spliceosome (complex B), which is then converted into a catalytically active complex B* by removing U1 and U4. Two subsequent transesterification steps and conformational and compositional rearrangements form complex C. Exon ligation follows, and mature mRNA is formed (11).

Splicing may be constitutive or alternative. Constitutive splicing events usually generate single transcripts from the pre-mRNAs. By contrast, alternative splicing events occur when at least two 5’ss or 3’ ss compete (12). Constitutive splicing usually occurs in the presence of strong splice sites containing consensus sequences that are easily recognized by the spliceosome. However, alternatively spliced exons usually contain weaker splice sites that can only be recognized *via* the mediation of additional splicing regulatory elements (SREs).

### Alternative Splicing Regulation

Alternative splicing arises from specific exons/introns that may or may not be included in a mature mRNA transcript. The five basic alternative splicing patterns are exon skipping, mutually exclusive exons, alternative 5’ss, alternative 3’ss, and intron retention (12). Alternative splicing is controlled by *cis*-regulatory elements and *trans*-acting factors. Certain epigenetic factors, such as transcription, chromatin structure, DNA methylation, and histone modifications, may also affect SRE function. Depending on their relative positions and activities, *cis*-regulatory elements are classified as exonic splicing enhancers (ESE), intronic splicing enhancers (ISE), exonic splicing silencers (ESS), or intronic splicing silencers (ISS) (13). *Cis*-regulatory elements can recruit *trans*-acting splice factors (RNA-binding proteins or RBPs) either to promote or suppress the recognition of nearby splice sites. Therefore, various RBP categories, expression levels, and activities may influence the regulation of alternative splicing. Thus far, many RBPs have been found to modulate the splicing mechanism. These include serine/arginine-rich (SR) proteins, heterogeneous nuclear ribonucleoproteins (hnRNPs), neuro-oncological ventral antigens (NOVA) proteins, polypyrimidine track binding proteins (PTB), forkhead boxes (FOX) proteins, and others. Of these, the most extensively studied are the SR proteins and the hnRNPs (14).

SR proteins or SR-rich splicing factors (SRSFs) structurally resemble each other and are characterized by the RS domain [rich in arginine (R) and serine (S) residues] at the *C*-terminal and one or two RNA recognition motifs (RRMs) at the *N*-terminal (13). The SR protein is usually a positive splicing factor and it facilitates splice site recognition. When it binds the ESE, the SR protein recruits U1 and U2 snRNPs and auxiliary factors, such as U2AF. In this way, it induces spliceosome assembly followed by exon inclusion (15). SR protein mutation or aberrant post-translational modification may lead to various diseases. Correct SR protein phosphorylation is vital in the regulation of the splicing event and alterations in subcellular localization, activity, and stability that may affect protein–protein and protein–RNA interactions (16). By contrast, hnRNPs are usually considered negative splicing factors that inhibit splice site recognition. For example, hnRNP blocks spliceosome access to the polypyrimidine tract by binding the ESS and promoting exon exclusion.

## Alternative Splicing in Various Aspects of Immunity

Autoimmune diseases are a subset of conditions rising from abnormal immune responses to a functional body part. Currently, specific candidate genes of autoimmune disease have been identified in multiple GWAS. Moreover, various alternative splicing events have been found in adaptive- and innate immune signaling-related genes. Elucidation of the mechanism of immune response related to autoimmune disease pathogenesis is essential for further investigations into alternative splicing regulation. In this section, we focus on the physiological and pathological roles of alternative splicing in immunity, and attempt to figure out common patterns of splicing regulation in different autoimmune diseases.

### Gene Categories Involved in Alternative Splicing in Autoimmunity

Alternative splicing is a pivotal mechanism widely acknowledged to regulate gene expression and promote protein diversity. The maintenance of functional immune responses requires protein diversity and flexibility, which are essential in alternative splicing regulation. Many genes involved in innate or adaptive immune signaling undergo varying degrees of alternative splicing. Ergun et al. determined by RNA sequencing and microarray that alternatively spliced isoforms occur in 60% of all T- or B-lymphocyte genes (17). Based on spliced gene functions, alternative splicing may affect the physiological function of the immune system in different ways.

#### Cell Surface Receptors

Cell surface receptors constitute a subset of transmembrane proteins that participate in signal transduction by receiving extracellular molecules. CD44, CD45, CD85, TCRζ, CTLA4, and others have alternative spliced isoforms. These structural variants have distinct activity and stability and may affect ligand binding and disrupt normal signaling. For example, a short 3’ untranslated region (UTR) isoform of TCRζ was generated by intron removal. It was relatively less stable, affected TCR/CD3 complex assembly and expression, and limited T-cell activation (18).

#### Cytokines and Their Corresponding Receptors

Cytokine secretion and reaction are crucial processes in immunomodulation. Immune cell stimulation produces various cytokines and their isoforms. These may either be similar or different in terms of biological activity. The functions of these spliced variants have not been fully determined. In most cases, however, isoforms of IL-2, IL-4, IL-6, and others might act as antagonists to their corresponding wild types and block their activity (19). Alternative splicing of cytokine receptors usually generates both membrane-bound and soluble isoforms with similar biological activity. However, soluble isoforms of IL-6R, TNFR2, and others cannot transmit signals through ligand binding and might act as inhibitors.

#### Intracellular Signaling

T-lymphocyte activation may result in subsequent signal-induced alternative splicing. In this way, they generate variant isoforms of downstream intracellular molecules, such as the Src family members Lck, Fyn, Syk, and others, that may disrupt normal signal transduction (6, 20). In innate immune cells, an isoform of the adaptor protein myeloid differentiation primary response 88 (MyD88) may inhibit downstream Toll-like receptor (TLR) signaling and limit innate immune response activation (21). Moreover, an isoform of TLR4 is associated with TNF-α and NF-κB downregulation (22).

#### Complement System

Activation of the complement system is an essential part of the innate immune response. Alternative splicing events have also been found in complement proteins and receptors, such as C2, CR1, CR2, and others (23). They have also been detected in complement regulatory proteins, such as membrane cofactor protein (MCP) (24).

### Splicing Regulatory Patterns in Autoimmunity

Basic alternative splicing patterns and its regulatory mechanisms are widely acknowledged. In autoimmunity, the disease-related pathogenesis varies from one to another. Here, we conclude the common features of alternative splicing in the pathological changes of autoimmune diseases.

#### Gene Mutations: The Most Common Cause of Alternative Splicing

Candidate single-nucleotide polymorphisms (SNPs) and other sequence mutations can directly change the coding region and result in aberrant alternative splicing. In most cases, SNPs are localized to non-coding sequences, which may disrupt consensus splicing sites or create novel alternative binding sites, thereby influencing the efficiency or selection mode of alternative splicing. Wang et al. reviewed 303 genes and 370 diseases with overall 2,337 splice mutation-disease entries from publication searching. Nearly 90% of the events resulted from point mutations (25). However, the regulatory mechanisms involved are not fully understood and they might affect alternative splicing in other ways.

#### SREs: Fundamental Regulatory Mechanism of Alternative Splicing

Epigenetic modification and changes in the levels of *cis*-regulatory elements and *trans*-acting factors can lead to different alternative splicing site selection and, therefore, variant transcripts. In human genome, there are numerous potential splice sites, known as pseudo-exons. It is relatively weaker than the consensus sequences during the recognition of snRNP. SREs can enhance or silence nearby splice sites, thereby promoting the diversity of selection between pseudo-exons and real exons. Weaker splice sites at the 5’ and 3’ end of intron may hamper the recognition of spliceosome, leading to specific intron retention (26). Epigenetic modifications bring additional different functional SREs and thereby influence the splicing process regulation.

#### Autoantigens: Prone to Be Alternative Spliced

Autoantigens are more frequently subjected to splicing control than non-autoantigens. Ng et al. compared the incidence of alternative splicing event in both randomly selected 45 autoantigens and 9,554 proteins from human genome. The results showed a 100% occurrence rate of alternative splicing in autoantigen transcripts, whereas 42% rate in the other group (27). Spliced isoforms have been found in various disease-specific autoantigens, such as multiple sclerosis (MS)-related autoantigen myelin oligodendrocyte glycoprotein (MOG) and transformation of myelin proteolipid protein (PLP), type 1 diabetes mellitus (T1DM)-related autoantigen islet cell Ag 512 (IA-2), Sjögren’s syndrome (SS)-related autoantigen SS-B/La and SS-A/Ro, Graves’ disease (GD)-related autoantigen thyroid peroxidase (TPO), and others (28–33). Moreover, some nucleus antigens, such as RA33, Sm, U1 snRNP, Jo-1, NOR-90, histone, DNA Topo 1, ribosomal P2, and others, also undergo some level of alternative splicing (27, 34–36). The increased noncanonical splicings and posttranslational modifications may give rise to novel untolerized antigenic epitopes that could enhance the immunogenicity of the autoantigens. Moreover, spliced isoforms with variation at the linker region can influence ligand binding and subsequent downstream signaling. Some specific autoantigens, such as U1 snRNP, Sm may even affect the splicing procedures. Assessment of these antigens or antibodies is of great importance in the diagnosis and monitor of autoimmune diseases.

#### Soluble Isoforms: May Serve as Predictors or Natural Inhibitors

High level of soluble isoforms, such as sIL-6R, sIL-7R, sgp130, sCD44v5, sCD44v6, and sCTLA4, are observed in most autoimmune diseases. Unlike membrane-bound isoforms, soluble isoforms are readily detected in peripheral blood. In certain cases, their levels there indicate disease severity and activity. Hence, they might be useful in disease monitoring. By contrast, soluble isoforms can also be natural competitive inhibitors to membrane-bound isoforms. Therefore, the former block downstream signaling pathways and are potential therapeutic targets.

We attempted to identify the foregoing common splicing modulation patterns in different autoimmune diseases. We focused on systemic lupus erythematosus (SLE) and rheumatoid arthritis (RA). SLE is associated with autoimmunity induced by immune complexes (ICs), whereas RA is related to cytokine-mediated inflammation. We also explored spliced isoforms with potential diagnostic or therapeutic value.

## Spliced Isoforms in SLE

**Figure 2:**
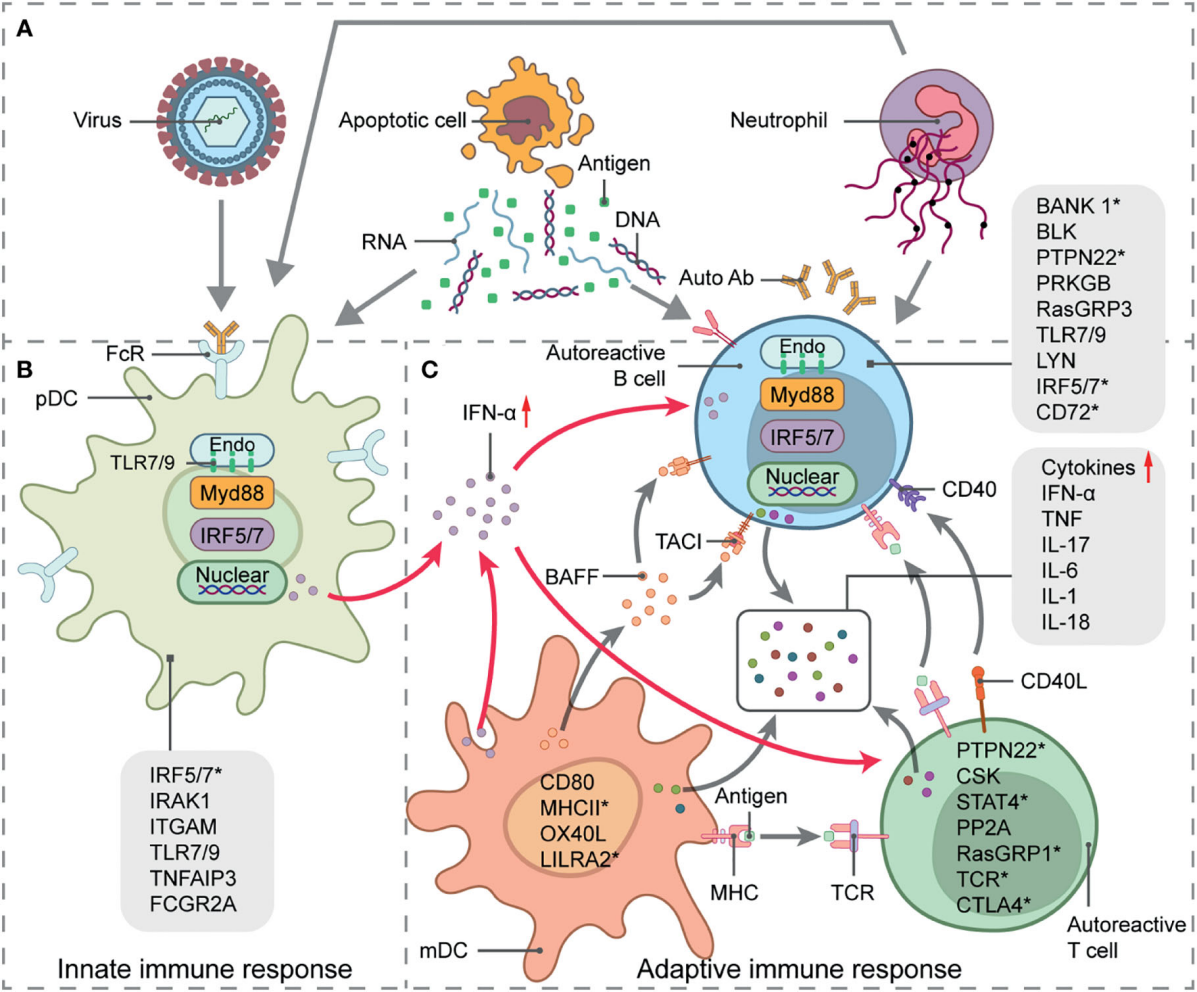
Systemic lupus erythematosus (SLE) genetics and pathogenesis. Schematic picture of SLE immunopathogenesis and alternative splicing involved in signaling pathways-related genes. **(A)** Source of autoantigens initiating innate and adaptive responses in SLE include viruses, apoptotic material, and neutrophil extracellular trap (NET) formation. **(B)** pDC activation, antigen presentation, and IFN-α secretion. **(C)** IFN-α signaling pathway stimulates T- and B-cell activation, which leads to autoantibody and proinflammatory cytokine production and eventual local tissue injury. Straight arrows represent stimulation and curved arrows represent target process activation or production. SLE-related susceptibility genes are indicated in immune signaling steps. Genes with * undergo alternative splicing.

SLE is a chronic autoimmune disease of unknown etiology. It more frequently affects women in their reproductive age. It is characterized by nuclear autoantigen and IC formation leading to organ-specific or systemic inflammation and tissue damage. Both innate and adaptive immune responses participate in SLE development (**Figure 2**). Viruses, excess apoptotic material, and neutrophil extracellular trap (NET) formation may be autoantigen sources. These are recognized by plasmacytoid dendritic cells (pDCs), which, in turn, activate innate immunity. A malfunctioning type I interferon (IFN) signaling pathway is an important clue in SLE as it plays essential roles in dendritic cell (DC) differentiation and maturation, the activation of autoreactive T cells and T cell-stimulating autoreactive B cells, and multiple autoantibody production. Along with several proinflammatory cytokines and chemokines, autoantibodies contribute to local tissue injury by immune complex deposition or complement activation. However, genetic factors increase the relative risk of SLE as well. Earlier GWAS identified over 50 genetic loci associated with SLE (37–40). Some of these might influence splicing. Furthermore, mutations and disruptions in the splicing machinery may also cause alternative splicing. Factors and conditions that increase SLE susceptibility have also been reported in previous studies.

In the following section, we address several of the best-studied examples of alternative splicing that affect both the adaptive- and innate immune signaling-related genes associated with SLE. We also summarize the roles of transcription factors in alternative splicing regulation.

### IRF5

IRF5 is a transcription factor belonging to the IRF family. IRF5 mainly regulates interferon expression by activating the innate immune response to viruses or inflammatory stimuli. Moreover, IRF5 modulates cellular maturation, differentiation, and apoptosis as well as proinflammatory cytokine generation (41). Both innate and adaptive immune signaling activate IRF5 *via* the TLR and MyD88 pathways (42). IRF5 overexpression is associated with clinical SLE progress. The human *IRF5* gene has nine coding exons and at least four non-coding alternative exons designated 1A, 1B, 1C, and 1D (41, 43). By differential selection of the coding or non-coding alternative exons, human *IRF5* could theoretically generate multiple distinct functional isoforms. Some of these have been identified and were designated V1–V11. The spliced variants generated *via* the inclusion of alternative exon 1B (v2, v9, and v10) are strongly linked to IRF5 upregulation and SLE susceptibility. However, genetic polymorphisms in the human *IRF5* locus disrupt normal splicing and increase the risk of aberrant spliced variant formation. The distinct haplotypes of four functional SNPs increase susceptibility to SLE and other autoimmune diseases. For example, the T allele of rs2004640 creates a novel 5’ss at the intron-exon boundary of alternative exon 1B. Thence, exon 1B is included in transcript production. The G allele of rs10954213 destroys the polyadenylation (polyA) site in the 3’ UTR region of exon 9. This mechanism arrests transcription and results in a relatively long, unstable transcript (44). A 5-bp (CGGGG) indel (insertion/deletion) located in the proximal promoter creates a novel binding site for the Sp1 transcription factor, which leads to alternative splicing (45). Furthermore, 30-bp indel polymorphisms in exon 6 select two alternative 3’ss. Insertion of these 3’ss produces V5 and V6 isoforms, whereas their deletion generates V1 and V4 isoforms (41). These isoforms are structurally and functionally distinct and have different expression levels. Hence, they influence IRF5 signaling and its downstream immune responses.

Murine IRF5 (MuIRF5) shares approximately 87% amino acid sequence homology with human IRF5. Different from the heavily spliced human IRF5, MuIRF5 primarily generates a full-length transcript. Nevertheless, Paun et al. have detected a splicing variant of IRF5 from the bone marrow of C57BL/6J mice, termed as BMv, which contains a deletion of the entire exon 5 and part of exon 4, 6 (46). In the MyD88 activated innate immune signaling, BMv serves as a relatively weaker activator to the downstream IFNA4 promoter, compared to the full-length transcript.

### LILRA2

Leukocyte immunoglobulin-like receptors (LILRs) are also known as CD85. This family of leukocyte receptors has 13 members and includes the two pseudogenes LILRP1 and LILRP2. LILRA2 is a stimulatory receptor that delivers positive signals and is widely expressed on B cells, dendritic cells, monocytes, macrophages, and a subset of natural killer (NK) cells. LILRA2 cross-links with the immunoreceptor tyrosine-based activation motif (ITAM) of Fc-γ receptor (FcRγ) and induces calcium mobilization and subsequent signaling activation (47). Genetic polymorphisms of human *LILRA2* were associated with increased SLE susceptibility in Japanese populations (48). The SNP rs2241524 G>A at the intron 6–exon 7 junction disrupts the consensus splicing acceptor motif and interferes with normal splicing. This mechanism results in a novel transcript with an in-frame deletion of three amino acids (Ala-Ser-Leu) at position 419–421 in the linker region (48). The impact of this spliced variant (Δ419–421 isoform) on ligand binding is unknown.

### BANK1

The B-cell scaffold protein with ankyrin repeats 1 (*BANK1*) gene encodes a scaffold adaptor protein expressed primarily in B cells. Stimulation of B-cell receptors (BCRs) induces tyrosine phosphorylation in BANK1, which then amplifies B-cell signaling by facilitating the activation of downstream signal molecules, such as Src-family kinases, the inositol 1,4,5-trisphosphate receptor (IP3R) calcium channel, phospholipase Cγ2 (PLCγ2), protein kinase C β (PRKCB), and Ras (49, 50). *BANK1* variants have been linked with susceptibility to multiple autoimmune diseases, such as SLE, RA, and SS (51–53). Kozyrev et al. analyzed BANK1 cDNA and identified a novel spliced transcript (50) designated Δ2 isoform because it lacks the entire exon 2. It encodes a protein without putative IP3R-binding or PH domains. This defect attenuates BANK1-mediated signaling. The Δ2 isoform is also detected from chimpanzee and mouse spleen. Three SNPs (rs10516487 G>A in exon 2, rs17266594 T>C in intron 1, and rs3733197 G>A in exon 7) associated with SLE might also influence Δ2 isoform splicing. The rs17266594 is localized to a putative splice BP of exon 2. This mutation affects relative splicing efficiency and upregulates the Δ2 isoform. The rs10516487 is associated with a decrease in exon 2 splicing (54). The precise role of BANK1-mediated signaling downregulation in SLE is unclear, but it may participate in SLE pathogenesis as B cells have vital functions in autoimmunity.

### RasGRP1

Ras guanyl releasing protein 1 (RasGRP1) is a guanine-nucleotide-exchange factor that is first cloned in the brain and is then highly expressed on T cells (55). *RasGRP1* is linked to autoimmunity. RasGRP1 deletion may lead to a deficiency of thymocyte differentiation or T-cell development in mice (56). In humans, RasGRP1 deficiency may interfere with T-cell and B-cell proliferation and activation, thereby causing various autoimmune disorders (57). T-cell receptor (TCR) stimulation drives protein tyrosine kinase activity and diacylglycerol (DAG) binding, which, in turn, recruit son of sevenless (SOS) to the plasma membrane and activate Ras (58, 59). RasGRP1 participates in TCR-induced signaling and activates Ras. RasGRP1 consists of a DAG-binding domain and two calcium-binding EF hands. Yasuda et al. identified 13 new spliced human RasGRP1 transcripts generated by alternatively including or deleting exons 5–17. These novel transcripts constitute an isoform family (splice variants A–M) (59). Nine of these were translated into truncated nonfunctional RasGRP1 isoforms because of a premature translation termination codon. The remaining four, however, did not cause any frameshift abnormalities or induce early translation termination. Compared with normal individuals, SLE patients lack the functional RasGRP1 isoform and have elevated levels of defective variant isoforms.

Splicing factors, such as SRSF1, regulate alternative splicing. The RasGRP1 transcript lacking exon 11 (splice variant A) is the type most observed in the T cells of SLE patients. SRSF1 directly binds the exon 11 of RasGRP1 mRNA, thereby promoting exon 11 inclusion. Kono et al. found that compared with the T cells of healthy individuals, those of SLE patients presented with significant SRSF1 downregulation. SRSF1 knockdown in healthy human T cells *via* small interfering RNAs (siRNAs) increased the splice variant A:normal RasGRP1 isoform ratio and downregulated RasGRP1 (60). Moreover, hnRNP-K protein was correlated with RASGRP1 expression and extracellular signal-regulated kinase (ERK) phosphorylation (61).

### TCRζ

The TCRζ chain is involved in TCR/CD3 complex assembly and surface expression (62). The cytoplasmic domain of TCRζ includes three ITAMs that are phosphorylated when TCR is activated. This mechanism leads to the recruitment of other signaling molecules and, in turn, the phosphorylation or activation of downstream signaling transduction molecules (63–65). T cells are central to SLE pathogenesis. In SLE, T cells display multiple abnormalities in their antigen‐mediated signaling. Recent studies showed that SLE patients had lower TCRζ mRNA expression levels than healthy subjects (66–68), possibly because of low transcription activity, splice variant generation, and other factors (69, 70). Alternative splicing generated a series of transcript variants of TCRζ, including TCRη (exon 8 replaced by exon 9), TCRι (exon 8 replaced by exon 10), an isoform lacking exon 7, and another with a short 3’UTR (69, 71–74). Similar variation of TCRζ transcript also exists in animal models. For example, TCRη contains completely uniform alternative exon 8 selection to human, and is observed in both mice and swine, while TCRι is observed in mice (75, 76). TCRη and TCRι are natural isoforms with normal function, whereas the other two are relatively unstable, more readily degraded than their full-length transcript counterparts, and detected exclusively in SLE patients. Variant isoforms affect the assembly of the TCR/CD3 complex and its expression on the cell surface. In addition, it changes the Treg/Th17 balance. Consequently, they attenuate normal signaling and result in T-cell dysfunction and insufficient IL-2 production (77–79).

A previous study demonstrated that epigenetic modification or changes in the levels of splicing factors may affect the mechanism regulating alternative splicing. In human T cells, SRSF1 can bind the 3’UTR region of TCRζ mRNA, thereby favoring the expression of TCRζ isoforms with full-length 3’UTR over unstable short ones (80). SLE patients usually present with relatively low SRSF1 levels and enhanced SRSF1 ubiquitination (81, 82). SRSF1 may increase total TCRζ mRNA expression, whereas SRSF1 knockdown may decrease it. The specific mechanism involved in SRSF1 regulation remains to be determined. However, SRSF1 might be a suitable therapeutic target for the restoration of normal T-cell function.

### Other Genes

Many studies have identified numerous loci of disease susceptibility-related genes associated with certain specific splicing patterns. In addition to the foregoing gene loci, there are several less common spliced variant isoforms related to SLE susceptibility.

CD72 is an inhibitory receptor expressed mainly on B cells. The *CD72* SNP affects normal splicing and generates a spliced isoform lacking exon 8 (CD72Δex8) that modulates antibody production. Polymorphisms of the Complement C3d Receptor 2 (*CR2*) gene also regulate alternative splicing. A haplotype formed by minor CR2 SNP alleles (rs17615 and rs1048971 in exon 10 and rs4308977 in exon 11) might affect splicing efficiency and significantly lowers the risk of SLE (83). The SNP of *CR2* known as rs3813946 is located in the 5’UTR and may also influence *CR2* transcription. CTLA4, also known as CD152, is a protein receptor expressed mainly on T cells. Variants of human *CTLA4* are associated with a wide range of autoimmune diseases, including SLE, RA, T1DM, GD, and MS. Alternative splicing of human *CTLA4* generates various transcripts, such as a membrane-bound form (mCTLA4), a soluble form (sCTLA4), and four other rare transcript types. Al Fadhli et al. reported that patients with SLE or RA express neither mCTLA4 nor sCTLA4 (84). sCTLA4 may interfere with the B7:CTLA4 interaction and block negative signals. However, the roles of various CTLA4 isoforms in autoimmune disease mechanisms remain to be clarified and additional studies are required to explore these signaling pathways.

## Spliced Isoforms in RA

**Figure 3:**
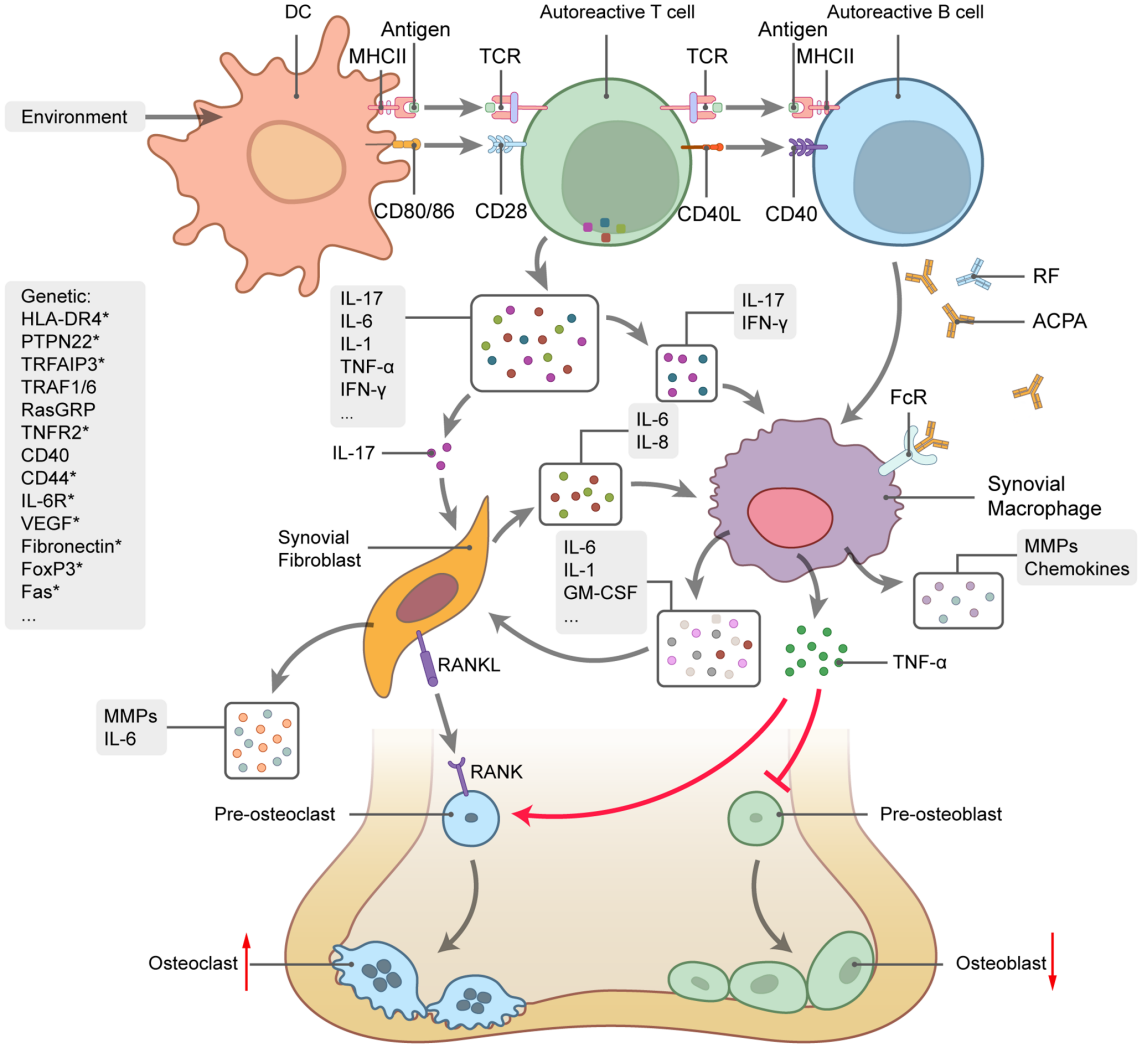
Rheumatoid arthritis (RA) genetics and pathogenesis. Schematic picture of cytokine-mediated bone inflammation and destruction in RA. Genetic predisposition and environmental factors initiate the autoimmune response. Interactions between TCR and MHCII-antigen plus co-stimulation of the CD28-CD80/86 pathway trigger activation of T cells by DCs along with substantial cytokine production, B-cell activation, and autoantibody generation. IL-17 stimulates synovial fibroblast and macrophage proliferation and activation and promotes the secretion of proinflammatory mediators, such as TNF‐α, IL-1, IL-6, and GM-CSF. TNF-α regulates the balance between bone formation and destruction by promoting osteoclast precursor transformation to osteoclasts and inhibiting osteoblast precursor transformation to osteoblasts. Osteoclast differentiation also depends on the interaction between RANK and its ligand. Curved arrows represent target process activation and bar-headed lines represent target process inhibition. Straight arrows represent changes in quantity. Several RA-related genes involved in RA pathogenesis are listed on the left side of the picture. Genes with * undergo alternative splicing.

RA is a chronic autoimmune disorder characterized by persistent inflammation of the synovial joints. RA eventually results in cartilage and bone destruction. About 50% of all RA incidences are associated with genetic factors, such as the predisposing genotypes HLA, PTPN22, and STAT4. Lifestyle factors, such as smoking, obesity, and vitamin D deficiency, can also trigger RA onset (85). Disequilibrium of the respiratory mucosa is an initial sign of RA development and subsequently leads to immune activation, local inflammation, and the production of autoantibodies, including those against citrullinated protein antigens (ACPA) and rheumatoid factor (RF) (**Figure 3**) (86). Activated lymphocytes and autoantibodies migrate to the joints where they induce the production of proinflammatory chemokines and cytokines, such as TNF‐α, IL-1, IL-6, and IL-17. In turn, these facilitate the recruitment and activation of other immunocytes. IL-17 produced by helper T (Th17) cells stimulates the activation and proliferation of synovial fibroblasts and macrophages, which eventually affect the balance between osteoclasts and osteoblasts and ultimately lead to bone destruction (87).

Previous GWAS identified over 100 genetic loci encoding immune regulatory factors linked with RA. Moreover, several variant isoforms were identified among some of the candidate genes. In the following section, we review the major spliced gene transcripts participating in RA pathogenesis.

### CD44

CD44 is a cell-surface glycoprotein involved in cell survival, proliferation, migration, and traffic signal transmission, cell–cell interactions, and programmed cell death (88, 89). *CD44* comprises 10 constant and 10 variant (v1–v10) exons inserted between the 5’ and 3’ ends (90). Standard CD44 (CD44s) is the most ubiquitous form. It contains only constant exons and is expressed mainly on hematopoietic cells. Alternative splicing of the variant exons and *N*-glycosylation, *O*-glycosylation, and glycosaminoglycanation lead to numerous variant isoforms (CD44v). However, only a few dozen of these have been identified thus far (91). CD44 is closely related to multiple autoimmune disorders, including RA, SLE, MS, and inflammatory bowel disease (IBD). Compared with osteoarthritis patients, RA patients have relatively higher synovial CD44s, CD44v4, CD44v6, and CD44v7–8 transcript levels (92, 93). CD44v3 and CD44v6 are associated with increased tumor cell migration and invasion because they add adhesion and MMP docking domains. A similar mechanism occurs in RA fibroblast-like synoviocytes (FLS), and it augments their ability to invade Matrigel (94). Elevated serum levels of soluble CD44 (sCD44) variant isoforms, such as sCD44v5 and sCD44v6, have been detected in RA patients. Serum sCD44v5 is correlated with inflammation and could serve as a predictive factor in responses to RA therapy (95, 96).

### TNFR2

Tumor necrosis factor (TNF)-α is a pleiotropic proinflammatory cytokine implicated in inflammation, cell proliferation, differentiation, and apoptosis (97). TNF-α is one of the most potent proinflammatory cytokines in RA. TNF signaling is mediated by the receptors TNFR1 and TNFR2. Both of these belong to the TNFR superfamily and have high affinity for TNF-α (98). TNFR1 is widely expressed on most cells and is both proinflammatory and apoptotic. TNRF2 expression is limited mainly to endothelial cells and certain immunocytes and participates in anti-inflammatory processes and cell proliferation (99, 100). Proteolytic cleavage produces soluble forms of TNFR (sTNFR). Elevated serum sTNFR is observed in certain infections, cancers, and autoimmune disorders, such as RA, SLE, and SS (101, 102) Recent studies identified an alternatively spliced TNRF2 isoform lacking exons 7 and 8 that encode transmembrane and cytoplasmic domains (DS-TNFR2) (103, 104). TNRF2 is a soluble receptor that acts as an antagonist in TNF‐α-mediated signaling and blocks apoptosis (104). Cañete et al. demonstrated that alternatively spliced DS-TNFR2 constitutes the majority of the sTNFR2 in RA patients, and serum sTNFR2 is closely associated with RA activity and severity (101, 103).

### IL-6R and gp130

IL-6 is a pleiotropic cytokine that is crucial in chronic inflammation and multifactorial autoimmune disorders, such as RA, IBD, MS, and Castleman’s disease. IL-6 overexpression may contribute to clinical RA symptoms. In classic signaling, IL-6 binds the cognate ligand-binding subunit (IL‐6R, CD126). This mechanism results in the recruitment of the signal-transducing components gp130 and CD130, which, in turn, promote anti-inflammatory responses (105). Gp130 is widely expressed on most cell types, whereas IL-6R is detected on only a few specific cells, such as monocytes, macrophages, neutrophils, lymphocytes, and hepatocytes (106). IL-6R can exist in both membrane-bound (mIL-6R) and soluble (sIL-6R) form. About 90% of all sIL-6Rs are transformed from mIL-6R *via* limited proteolysis of metalloproteinase domain-containing protein 17 (ADAM17) (106, 107). The SNP rs8192284 A > C localized to the proteolytic cleavage site of exon 9 was associated with an increase in mIL-6R shedding (108, 109). The exclusion of exon 10 encoding the transmembrane region and IL-6R mRNA splicing generate a minor proportion of the sIL-6R (110). The loss of mIL-6R disrupts classic IL-6 signaling whereas formatted sIL-6R is biologically active. By binding IL-6, the sIL‐6R/IL‐6 complex can interact with gp130, which leads to intracellular signaling in cells that do not otherwise express IL-6R (111–113). This process is known as IL-6 trans-signaling and it induces proinflammatory responses (114). Upregulated sIL-6R has been observed in both RA and osteoarthritis. Hence, sIL-6R is implicated in joint inflammation and destruction (115). IL-6R inhibitors, such as tocilizumab and sarilumab, target binding to mIL-6R and sIL-6R. Therefore, they block both classic IL-6 signaling and trans-signaling. Their efficacy in RA treatment has been demonstrated (116). Recently, soluble gp130 (sgp130) variants arising from alternative splicing or polyadenylation have been identified. Sgp130 variants are considered natural trans-signaling inhibitors. Their three known isoforms are full-length sgp130, sgp130-RAPS, and sgp130-E10 (117, 118). Fc-dimerized sgp130 protein (sgp130Fc) blocks IL-6 trans-signaling and has been evaluated in the treatment of various inflammatory disease models. It is currently undergoing clinical trials as a drug candidate for IBD (119) and is also a potential therapeutic agent for RA.

Along with the foregoing soluble isoforms, alternative splicing generates others associated with RA such as sIL-7R (excision of exon 6 containing the transmembrane domain) (120), sCD137 (lacking the 414–545 nucleotide region and characterized by a frameshift) (121), sCD1d (excision of exons 4 and 5 containing the transmembrane domain) (122), and FasΔTM (excision of exon 6) (123).

### VEGF

Vascular endothelial growth factor (VEGF) is a highly specific vascular endothelial cell mitogen. *In vivo*, VEGF induces microvascular permeability and plays central roles in regulating angiogenesis and tumor neovascularization (124). Prior studies demonstrated that VEGF-mediated angiogenesis also occurs in the synovial tissues of RA patients. In RA, VEGF polypeptide is highly expressed on synovial macrophages, fibroblasts, vascular smooth muscle cells, and synovial lining cells and in synovial fluids (125, 126). Human *VEGF* comprises eight exons and seven introns. Alternative selection of exons 6–8 and VEGF mRNA splicing generate the variant transcripts VEGF−A121, VEGF−A145, VEGF−A165, VEGF−A189, and VEGF−A206. These are generically designated VEGF−Axxx where xxx indicates the number of amino acids in the mature protein (127). VEGF121 and VEGF165 are the only VEGF-spliced isoforms expressed in RA. VEGF165 has 15 basic amino acids in 44 residues encoded by exon 7 and it binds heparan sulfate. By contrast, VEGF121 lacks this region and its diffusibility differs from that of VEGF165 (128).

### Fibronectin

Fibronectin (Fn) is a multifunctional glycoprotein that is widely expressed in the extracellular matrix, plasma, synovial fluid, and other fluids. From a single gene, alternative Fn mRNA splicing generates multiple distinct isoforms that play vital roles in embryonic development, inflammation, wound repair, malignant metastasis, and thrombosis. Recognized alternative splicing sites include extra domain A (EDA), extra domain B (EDB), and type III connecting segment (IIICS) (129, 130). EDA and EDB each encode a single homology type III repeat (131). RA patients present with elevated synovial EDA(+)FN. This marker is correlated with accelerated rheumatoid joint destruction. The EDA(+)FN synovial fluid level is proportional to the severity of RA signs and symptoms and is promising as a predictor of RA progression (132). In animal model, Gondokaryono et al. found that EDA(+)FN might induce joint swelling in a mast cell TLR4-dependent manner by injecting EDA(+)FN to mast cells that lacked W/Wv mice (133). Removal of EDA(+)FN from the plasma can efficiently and effectively mitigate RA symptoms. EDA(+)FN cryofiltration and selective EDA(+)FN adsorbents have been developed for this purpose (134). Furthermore, heparin has high affinity for FN and can selectively bind and remove EDA(+)FN from the plasma.

## Alternative Splicing in Other Autoimmune Diseases

Autoimmune diseases other than SLE may also be the result of aberrant alternative splicing. For example, alternative transcripts of disease-specific antigens could serve as novel antigenic epitopes affecting ligand binding and, by extension, autoimmunity. High level of a shorter splice variant of PLP, DM20 lacking exon 3B, in the thymic epithelial cells can induce the T-cell tolerance to all epitopes of the PLP protein (135). In an animal model of MS, SJL/J mice lack thymic DM20 and thereby experience loss of the central tolerance to this epitope and increased susceptibility to MS (28). Distribution and expression of IA-2 isoforms can influence immune responsiveness to specific epitopes. Islets express full-length IA-2 and two spliced isoforms, IA-2Δ13 (lacks exon 13) and IA-2Δ14 (lacks exon 14), whereas thymus and spleen exclusively express IA-2Δ13. Hence, IA-2 could serve as a target of T1DM (30). Moreover, SS-B/La isoform (exon 1 exchange) might be a target of Sjögren’s syndrome (SS) (31). **Table 1** lists several autoimmune diseases, such as MS, T1DM, IBD, MG, SS, SSc, and GD, which are associated with alternative splicing. Diagnostic or pathologic roles of spliced isoforms in relevant autoimmune diseases are also cited.

**Table 1:** Alternatively spliced genes and isoforms in related autoimmune diseases.

Autoimmune diseases	Gene	Isoform (alternatively spliced site)	Functional outcome	References
Systemic lupus erythematosus	IRF5	V1-11 (alternative 5’ss)	Elevated expression of IRF5 and IFN	(136)
	LILRA2	Δ419–421 (SNP in intron 6–exon 7 junction)	/	(48)
	BANK1	Δ2 (lacks exon 2)	Unable to transmit downstream signaling	(50)
	RasGRP1	Splice variants A-M (alternatively splicied exons 5–17)	Nonfunctional isoforms and blockage T-cell maturation	(59)
	TCRζ	Splice variants (lacks exon 7; a short 3′ UTR)	Decrease TCR signaling and disrupt Treg/Th17 balance	(69, 71–73, 137)
	CD72	CD72Δex8 (lacks exon 8)	Accumulated in the endoplasmic reticulum and does not regulate BCR signaling	(138, 139)
	CR2	A short isoform (spliced exon 11)	/	(83)
	CTLA4	sCTLA4 (lacks exon 4)	Bind to B7 and interfere with B7:CD28-mediated costimulation of T-cell responses	(84)
			Biomarker of disease activity	
Rheumatoid arthritis	CD44	CD44v3, v4, v5, v6, v7–8 (alternatively spliced v1–10 and differential glycosylation)	Increase invasive capacity to matrigel	(93, 95)
			Biomarker of disease activity	
	TNFR2	DS-TNFR2 (lacks exons 7 and 8)	Antagonist of TNF-α signaling	(103, 104)
			Biomarker of disease activity and severity	
	IL-6R	sIL-6R (excision of exon 10)	Lead to IL-6 trans-signaling	(110)
	Gp130	sgp130	Antagonist of IL-6 trans-signaling	
	VEGF	VEGF121, 165 (alternatively spliced exons 6–8)	Angiogenesis	(127)
	Fn	EDA(+)FN (segments insert)	Biomarker of joint destruction	(129)
	IL-7R	sIL-7R (excision of exon 6)	Antagonist of synovial cell activation	(120)
			Biomarker of response to TNF blockade therapy	
	CD137	sCD137 (lack nucleotides 414 to 545)	/	(121)
	CD1d	sCD1d (excision of exons 4 and 5)	Lowers expression level and alters NKT cell function	(122, 140)
	Fas	FasΔTM (excision of exon 6)	Oligomerization can induce cell death	(123)
	IL-32	IL-32γ (SNP)	Amplify TNFα-mediated inflammatory cascade	(141, 142)
	MAP2K4	MAP2K4v2 (skipped exon 5)	/	(143)
	PTPN22	PTPN22.6 (lacks exons 5–9)	Lead to hyperactivation of T cells	(144)
			Biomarker of disease activity	
	FoxP3	FoxP3Δ2 (lacking exon 2)	/	(145)
	TNFAIP3	Four isoforms (insertion of intron 2, 4 or deletion of exon 4)	Induce persistent NF-κB activation	(146)
Multiple sclerosis	IL-7R	sIL-7R (skips of exon 6)	/	(147, 148)
	MOG	Various isoforms	Affect cellular localization and transport	(29)
			Interact with anti-MOG antibodies and thus prevent demyelination	
	PLP	DM20 (lacks exon 3B)	Disrupt the PLP/DM20 ratio in thymus and result in self-epitope rejection from central tolerance	(28, 135)
	IFNAR2	IFNAR2a,b,c (alternatively spliced exons 8)	/	(149)
	FoxP3	Foxp3-E2 (inclusion of exon 2)	Alter suppressive function of Treg cell	(150)
	PRKCA	Two isoforms (alternative spliced exon 3)	/	(151)
	CD45	Five isoforms (alternative spliced exons 4–6)	/	(152)
Type 1 diabetes mellitus	TAP2	Two isoforms (alternative spliced exon 11)	More antigens presented	(153)
	IA-2	IA-2Δ13, Δ14 (lacks exon 13 or 14)	New antigenic epitopes	(30)
	CTLA4	sCTLA4 (lacks exon 4)	Potentiate Treg cell function	(154)
	Bim	Bim S (inclusion of exon 4)	More potent pro-apoptotic activity	(155)
	Deaf1	Deaf1-Var1 (intron insertion between exons 6 and 7)	Inhibit Deaf1 transcriptional activity and decrease the expression of peripheral tissue antigens in lymph nodes	(156)
	FOXP3	Foxp3-E2 (inclusion of exon 2)	Alter suppressive function of Treg cell	(150)
	G6PC2	Various isoforms (lacks exons 2, 3, and 4)	Differential expression in pancreas and thymus and disrupt self-tolerance	(157, 158)
	Adora1	Adora1-Var (lacks exon 2)	Inhibitor of Adora1	(159)
Inflammatory bowel disease	NOD2	Various isoforms	Unresponsive receptors	(160)
	CD44	CD44v7 (alternatively spliced v7)	Promote colitis by preventing T-cell apoptosis	(161)
	IGF	IGF-IEc (alternative spliced exons 5–6)	Induce smooth muscle cell hypertrophy and contribute to intestinal stricture	(162)
	PTPσ	Isoform (skips of exons 9)	Lack Ig-like domain	(163)
Myasthenia gravis	CTLA4	sCTLA4, LCTLA4	Bind to B7 and interfere with B7:CD28-mediated costimulation of T-cell responses	(164)
	AChE	AChE-R (alternative 3′ss)	Enhance ACh hydrolysis and restore the balance between ACh and AChE	(165, 166)
Sjögren’s syndrome	SS-B/La	Exon 1’ SS-B/La (replace of exon 1)	New antigenic epitope	(31)
	SS-A/Ro	52β (skips exon 4)	New antigenic epitope	(32)
	OAS1	p46, p42, and p48 (SNP-related alternative 3′ss)	Less responsive to IFN stimulation	(167)
	BAFF	ΔBAFF (skips exon 3 or 4)	Inhibitor of BAFF and blockage B-cell maturation	(168)
Systemic sclerosis	VEGF	VEGF 165b	Inhibitor of VEGF and lead to insufficient angiogenesis	(169)
	IL-4	IL-4δ2 (skips exon 2)	Inhibitor of IL-4	(170)
	CTLA4	sCTLA4 (lacks exon 4)	Interfere with B7:CTLA4 interaction and block negative signals	(171)
			Biomarker of disease sevirity and activity	
Graves’ disease	TPO	TPO2, 4, 5, zanelli (lacks exon 10, 14, 8, and 16)	Trapped in the endoplasmic reticulum and enzymatically inactive	(33, 172)

## Therapeutic Strategy Targeting Alternative Splicing

Alternative splicing modulation has been applied in many different areas even though its underlying regulatory mechanisms are poorly understood. Prior studies have attempted to identify causes or potential therapeutic targets *via* disease-related alternative splicing analyses. A recently discovered structural splicing enhancer (SSE) promotes tumor cell invasion and metastasis by interacting with small nuclear ribonucleoprotein polypeptide A’ (SNRPA1) (173). Shen et al. reported that, in mice, inhibiting the spliceosome by knocking down 14 splicing factors with siRNAs could drive the transition from pluripotent to totipotent stem cells. Hence, this mechanism has great potential in stem cell therapy (174). Certain therapies targeting the regulatory mechanism of alternative splicing have already been approved for cancers and neurological disorders.

Splice-switching oligonucleotides (SSOs) are specialized antisense oligonucleotides (ASOs) targeting pre-mRNAs. They comprise a major validated approach towards modulating alternative splicing events. SSOs bind various functional splicing factors, promote or inhibit exon and intron inclusion or skipping, and participate in the generation of expected spliced isoforms. SSOs have been approved for the treatment of Duchenne’s muscular dystrophy (DMD), spinal muscular atrophy (SMA), and other conditions (175, 176). For example, Graziewicz et al. designed a series of SSOs intended to promote exon 7 skipping in mouse *TNFR2* mRNA. The SSOs upregulated the soluble TNFR2 isoform, which, in turn, naturally blocked TNF-α signaling (177). Therefore, SSO application might be a promising therapeutic avenue because TNF-α overexpression is a critical feature in RA immunopathogenesis. Furthermore, a synthetic 20-base ASO directed target at exon 2 of the AChE mRNA, known as Monarsen, is designed for the treatment of MG patients. According to a non-placebo-controlled phase Ib study of Monarsen, more than 90% of patients felt an improvement in symptoms (166, 178). Monarsen is currently in a Phase II study. Kinases and phosphatases in the serine-arginine protein kinase family (SRPKs) and CDC2-like family of kinases (CLKs), protein phosphatases, and certain small molecules can affect alternative splicing by modulating splicing factor activity or levels (8). Other technologies such as siRNAs and clustered, regularly interspaced, short palindromic repeat‐associated protein 9 (CRISPR‐Cas9) regulate alternative splicing events *via* gene editing. Moreover, targeted disease-related spliced variant degradation may help treat certain diseases. There is abundant theoretical evidence for numerous efficacious therapies targeting the regulatory mechanism of alternative splicing. Nevertheless, we lack empirical evidence for the effectiveness of these approaches in the management of autoimmune diseases. Thus, further research is required to validate the feasibility of alternative splicing manipulation.

## Conclusion and Prospective

Autoimmunity is closely associated with alternative splicing. Most candidate autoimmune genes may produce alternative isoforms. Though the present review is not exhaustive, it provides a fairly comprehensive synopsis of the most extensively studied alternative splicing events in autoimmune diseases, such as SLE and RA. Numerous variant mRNA transcripts and proteins were identified *via* literature searches. However, the actual number of functional isoforms might be less than anticipated because some of them were either untranslated or underwent premature translation because of the presence of a termination codon in the transcript.

As high-throughput sequencing continues to increase in depth and capacity, RNA sequencing (RNA-Seq) might play an important role in elucidating transcriptome and alternative splicing (179). Recently developed third-generation RNA-Seq has the advantage of long read length and can facilitate the analysis of directly spliced isoform structures as well as the discovery of thousands of novel distinct transcript products (180). Innovative technologies will deepen our understanding of the mechanisms regulating alternative splicing in various diseases and help develop new diagnostic and therapeutic tools for them.

## Author Contributions

FH and WL conceived and designed the study. PR, LL, and SC completed the literature review and wrote the first draft. PR and LL contributed to the design of figures and table. FH, WL, and JC revised the manuscript for intellectual content. All authors contributed to the article and approved the submitted version. We would like to thank all participating authors.

## Funding

This study was supported by the funds from the Primary Research & Development Plan of Zhejiang Province (2020C03034) to FH and the Project of Natural Science Foundation of Zhejiang Province (Q19H050030) to PR.

## Conflict of Interest

The authors declare that the research was conducted in the absence of any commercial or financial relationships that could be construed as a potential conflict of interest.

## Publisher’s Note

All claims expressed in this article are solely those of the authors and do not necessarily represent those of their affiliated organizations, or those of the publisher, the editors and the reviewers. Any product that may be evaluated in this article, or claim that may be made by its manufacturer, is not guaranteed or endorsed by the publisher.
