# Corrigendum: Alternative splicing: a new cause and potential therapeutic target in autoimmune disease

**DOI:** 10.3389/fimmu.2024.1513491

**Published:** 2024-11-13

**Authors:** Pingping Ren, Luying Lu, Shasha Cai, Jianghua Chen, Weiqiang Lin, Fei Han

**Affiliations:** ^1^ Kidney Disease Center, The First Affiliated Hospital, Zhejiang University School of Medicine, Hangzhou, China; ^2^ Key Laboratory of Nephropathy, Zhejiang Province, Hangzhou, China; ^3^ Institute of Nephropathy, Zhejiang University, Hangzhou, China; ^4^ Department of Nephrology, The First People’s Hospital of Wenling, Taizhou, China; ^5^ Institute of Translational Medicine, Zhejiang University of Medicine, Hangzhou, China

**Keywords:** alternative splicing, autoimmune disease, systemic lupus erythematosus, rheumatoid arthritis, therapy

In the published article, there was an error regarding the affiliation for author Shasha Cai. As well as having affiliation 4, she should also have “Kidney Disease Center, The First Affiliated Hospital, Zhejiang University School of Medicine, Hangzhou, China”.

The authors apologize for this error and state that this does not change the scientific conclusions of the article in any way. The original article has been updated.

